# Phase-of-care mortality assessment in cardiogenic shock due to end-stage heart failure

**DOI:** 10.1016/j.jhlto.2024.100077

**Published:** 2024-03-02

**Authors:** Hoong Sern Lim, Jorge Mascaro

**Affiliations:** a Institute of Cardiovascular Sciences, University of Birmingham, Birmingham, UK; b Queen Elizabeth Hospital Birmingham, University Hospitals Birmingham NHS Foundation Trust, Edgbaston, Birmingham, UK

**Keywords:** cardiogenic shock, heart failure, heart transplantation, mechanical circulatory support, multi-organ failure

## Abstract

End-stage heart failure-related cardiogenic shock (HF-CS) is associated with high risk of short-term mortality, but the causes and mode of death in HF-CS have not been described. This study aimed to (i) describe the causes/modes of death in patients with HF-CS based on the phases-of-care (Rescue-Optimization-Stabilization-Exit therapy), analogous to the phase-of-care mortality analysis, and (ii) assess the impact of the introduction of a standardized team-based care. We included 120 consecutive patients with HF-CS who underwent temporary mechanical circulatory support. The introduction of standardized team-based care reduced mortality at 6 months (36/63 (57%) vs 17/57 (30%), *p* = 0.003), but did not alter the distribution of phase-of-care mortality. There were fewer deaths following heart transplantation/left ventricular assist device therapy with standardized team-based care (6% vs 28%, *p* = 0.067) may be clinically relevant.

Mortality associated with cardiogenic shock (CS) remains high despite greater utilization of temporary mechanical circulatory support (tMCS). Understanding the modes of death could provide insights to guide management in CS. To our knowledge, no studies have reported on the modes of death in patients with end-stage heart failure-related CS (HF-CS) supported with tMCS devices.

We have previously described 4 therapeutic stages of CS (Recognize/Rescue – Optimization – Stabilization – de-Escalation/Exit therapy, i.e., ROSE), and that standardized team-based management at each stage improved 6-month survival in patients with HF-CS.^1^ The improvement in survival was related to a combination of standardized and earlier deployment of tMCS before severe multiorgan dysfunction and better outcomes from heart transplantation/durable left ventricular assist device (LVAD) therapy from meticulous optimization/stabilization.

This study builds on our previous work to describe the causes/phase-of-care mortality triggers in patients with HF-CS supported by tMCS and the impact of standardized team-based care on phase-of-care mortality.

## Methods

This single-center study included consecutive patients with HF-CS (i.e., known cardiomyopathy prior to CS presentation) from January 2012 to January 2023 who underwent tMCS (intra-aortic balloon pumps excluded). Patients were divided into 2 groups: group 1 from January 2012 to August 2018 and group 2 from September 2018 to January 2023 to coincide with the introduction of standardized team-based care in August 2018. Other causes of CS were excluded.

All deaths were discussed at our multidisciplinary governance reviews to determine the phase-of-care mortality analysis (POCMA). Older cases were reviewed retrospectively. This work has been approved by our institution as part of on-going service improvement and informed consent was waived. The POCMA is a recognized quality improvement tool to categorize the seminal event for mortality commonly used in cardiac surgery.^2^ The POCMA was modified for CS based on the 4 therapeutic phases:

i. Recognize and Rescue phase

– ≤48 hours of tMCS

ii. Optimization phase

– >48 hours from tMCS until criteria for “Stabilization” achieved

iii. Stabilization phase

– All of the following:
  a. Minimal/no vasoactive drugs (maximum of 1 agent with vasoactive inotrope score <10)
  b. Consistent improvement (>48 hours) in at least 2 organ systems without deterioration in any organ function based on clinical and/or laboratory assessment (e.g., liberation from mechanical ventilation, improvement in liver enzymes and platelet counts)
  c. Absence of complications requiring intervention (e.g., vascular repair) or planned tMCS reconfiguration
  d. Initiation of physical rehabilitation

iv. Exit and de-Escalation phase

– Up to 6 months from liberation from tMCS for recovery, heart transplantation, or durable LVAD implantation

### Statistical analysis

Parametric and nonparametric data are presented as mean and standard deviation or median and interquartile range, respectively; and between-group differences for continuous variables are analyzed by Student’s *t*-test and Mann-Whitney test. Statistical significance was inferred at a *p*-value <0.05 (Stata version 16.1, TX).

## Results

One hundred and twenty consecutive patients with HF-CS were included: 63 in group 1 and 57 in group 2. Patients in group 1 were more likely to be in INTERMACS profile 1 and had more severe organ dysfunction prior to tMCS (Table 1). There was a significant standardization in the use of tMCS in group 2, but the number of tMCS procedures (≥2tMCS in 20/63 vs 20/57 patients *p* = 0.331) and duration of tMCS to Exit therapy (13 (7-25) days vs 15 (7-34) days, *p* = 0.172) were comparable.

**Table 1:** Patient Characteristics at Baseline

Patient Characteristics	Group 1 (*n* = 63)	Group 2 (*n* = 57)	*p*
Age (years)	41 ± 4	45 ± 4	0.104
Males (*n*, %)	40 (64)	46 (81)	0.037
BMI (kg/m^2^)	26 ± 1	27 ± 1	0.574
Etiology (*n*, %)			0.018
DCM	52 (83)	37 (65)	
Ischemic	6 (10)	17 (30)	
Others	5 (7)	3 (5)	
HF > 1 year (*n*, %)	43 (68)	43 (75)	0.383
INTERMACS (*n*, %)			<0.001
Profile 1	46 (73)	9 (15)	
Profile 2	17 (27)	48 (84)	
Ventilated (*n*, %)	25 (40)	8 (14)	0.002
Inotropes			
≥3 drugs (*n*, %)	21 (33)	15 (26)	0.493
Epinephrine (*n*, %)	33 (48)	11 (19)	<0.001
Norepinephrine (*n*, %)	45 (71)	30 (53)	0.034
LVEF (%)	11 (10-15)	10 (10-15)	0.345
Severe MR (*n*, %)	17 (27)	16 (28)	0.894
Severe TR (*n*, %)	18 (29)	14 (25)	0.620
TAPSE (mm)	11 (10-12)	11 (10-13)	0.333
CVP (mm Hg)	19 (15-21)	17 (14-21)	0.450
Mean PA pressure (mm Hg)	37 ± 2	35 ± 2	0.467
Cardiac index (liter/min/m^2^)	1.73 (1.43-1.96)	1.46 (1.24-1.86)	0.004
MAP (mm Hg)	67 ± 1	70 ± 2	0.022
Bilirubin (μmol/liter)	55 ± 14	43 ± 9	0.173
Creatinine (μmol/liter)	150 (107-219)	123 (99-171)	0.021
Platelet (10^3^/μl)	126 (98-185)	170 (131-232)	0.004
pH	7.39 (7.21-7.46)	7.42 (7.37-7.46)	0.086
PF ratio (kPa)	39.4 ± 3.1	47.6 ± 4.9	0.004
BE	−5.4 ± 1.9	−1.8 ± 1.3	0.003
Lactate (mmol/liter)	6.2 (4.2-10.6)	3.5 (2.7-4.4)	<0.001
Total SOFA score	11 (8-13)	9 (7-10)	<0.001
Initial tMCS			0.044
ECMO	26 (41)	0 (0)	
ECMO + Impella CP	2 (3)	19 (33)	
Central ECMO	3 (5)	0 (0)	
Impella 5.0/5.5	0 (0)	4 (7)	
Centrimag BIVADs	32 (51)	34 (60)	
Number of tMCS devices			0.331
1	43 (68)	37 (65)	
2	19 (30)	16 (28)	
3	1 (2)	4 (7)	

BE, base excess; BIVAD, biventricular assist devices; BMI, body mass index; CVP, central venous pressure; DCM, dilated cardiomyopathy; ECMO, extracorporeal membrane oxygenation; HF, heart failure; LVEF, left ventricular ejection fraction; MAP, mean arterial pressure; MR, mitral regurgitation; PA, pulmonary artery; PF ratio, ratio of partial pressure of arterial oxygen to fractional inspired oxygen; SOFA, sequential organ failure assessment; TAPSE, tricuspid annular plane systolic excursion; tMCS, temporary mechanical circulatory support; TR, tricuspid regurgitation.

The Interagency Registry for Mechanically Assisted Circulatory Support (INTERMACS) profile was used to characterize the patients prior to tMCS.

Forty-two patients (35%) died on support before Exit therapy: group 1 26/63 (41%) vs group 2 16/57 (28%), *p* = 0.192. The remaining 77 patients bridged to Exit therapy: heart transplantation (28 (44%) vs 33 (58%), *p* = 0.141); durable LVAD (8 (13%) vs 7 (12%), *p* = 0.711); or recovery (1 (2%) vs 1 (2%), *p* = 0.809). Eleven patients died <6 months following Exit therapy: group 1: (8 and 2 deaths post-transplant and LVAD) and group 2 (1 death post-transplant). As a result, there were more deaths at 6 months in group 1 vs group 2 (36/63 (57%) vs 17/57 (30%), *p* = 0.003).

In both groups, over half the deaths occurred during the Rescue and Optimization phases. The majority were attributed to multiorgan failure (Supplementary Figure 1). There was no significant difference in the distribution of deaths between groups 1 and 2 (*p* = 0.277), although the reduction in deaths following Exit therapy in group 2 compared to group 1 was notable (6% vs 28%, *p* = 0.067).

## Discussion

Standardized team-based management has been shown to improve outcomes in CS in nonrandomized studies,^3^ but how this is achieved is unclear. We noted a significant reduction in 6-month mortality but no change in the distribution of phase-of-care triggers for mortality, suggesting that the benefits of standardized team-based management probably extended to all phases of CS. Indeed, reducing mortality through all phases of CS may be necessary for clinically significant improvement in CS survival (an Ishikawa fishbone diagram shown in Supplementary Figure 2). The corollary is that a single intervention or device, introduced in isolation may have only a modest impact on mortality in CS.

The implications of our findings are 3-fold:

First, earlier recognition and effective intervention may improve survival in HF-CS, as the majority of mortality triggers occurred during the early Rescue/Optimization phases. Multiorgan failure was the dominant cause of death, which at least partly reflects the failure to reverse organ dysfunction (i.e., “refractory shock”). “Refractory shock” was also the major cause of death in acute myocardial infarction-related CS.^4^ Earlier effective hemodynamic support may improve the likelihood of reversing hypoperfusion-related organ dysfunction.

Second, standardized team-based care was associated with a reduction in Exit Therapy deaths. Although not statistically significant, we view this finding as clinically significant because it appears to be especially modifiable through standardized team-based care and especially relevant to the utilitarianism of scarce donor organs.

Third, the development of additional complications, such as inflammatory response, thrombosis, and coagulopathy/bleeding (hemocompatibility-related complications^5^), further contributes to the development of multiorgan dysfunction syndrome. Indeed, hemocompatibility complications directly contributed to mortality despite achieving Stabilization. Minimizing hemocompatibility complications is a priority in tMCS.

### Study limitations

First, there are inherent biases associated with single-center observational studies. Second, we only included patients who had undergone tMCS, which may explain the differences in characteristics (e.g., younger age) and high incidence of heart replacement therapies compared to other studies of HF-CS.^6^ Third, our phase-of-care classification of deaths based on the ROSE phases has not been validated.

## Conclusion

Standardized multidisciplinary team-based care improved 6-month survival but did not affect the distribution of phase-of-care mortality triggers in patients with HF-CS. Reducing mortality at all phases of CS may be necessary for clinically significant improvement in CS survival.

## Disclosure statement

H.S.L. has received honoraria from Abiomed.

Funding and Acknowledgments: None.
