# Supplementary figures and images for: Phase-of-care mortality assessment in cardiogenic shock due to end-stage heart failure
Source: JHLT Open. 2024 Mar 2;4:100077. doi: 10.1016/j.jhlto.2024.100077 (PMC11935383; doi:10.1016/j.jhlto.2024.100077)

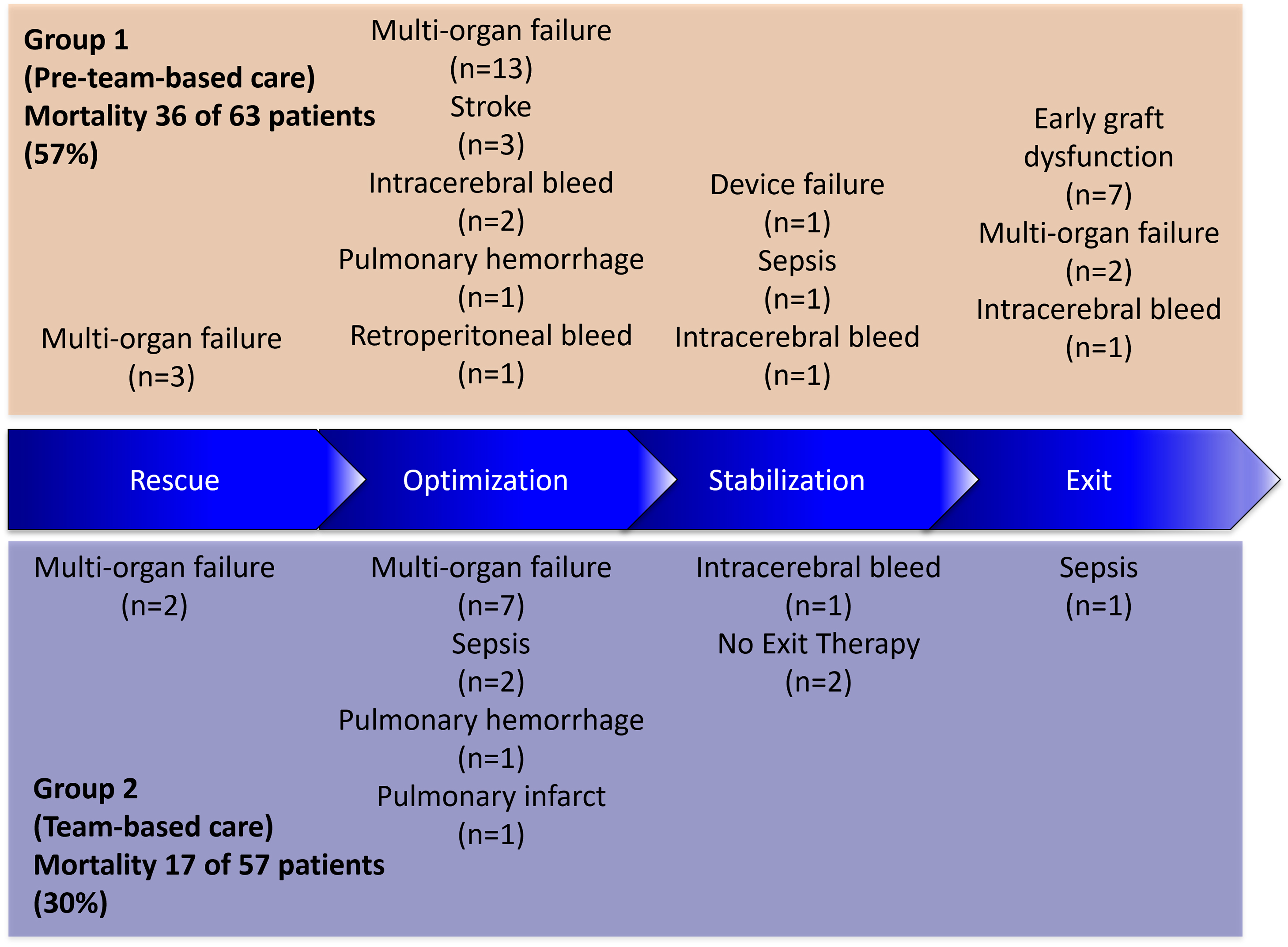

Supplement: Supplementary file 1 — Supplementary material [file mmc1.jpg]

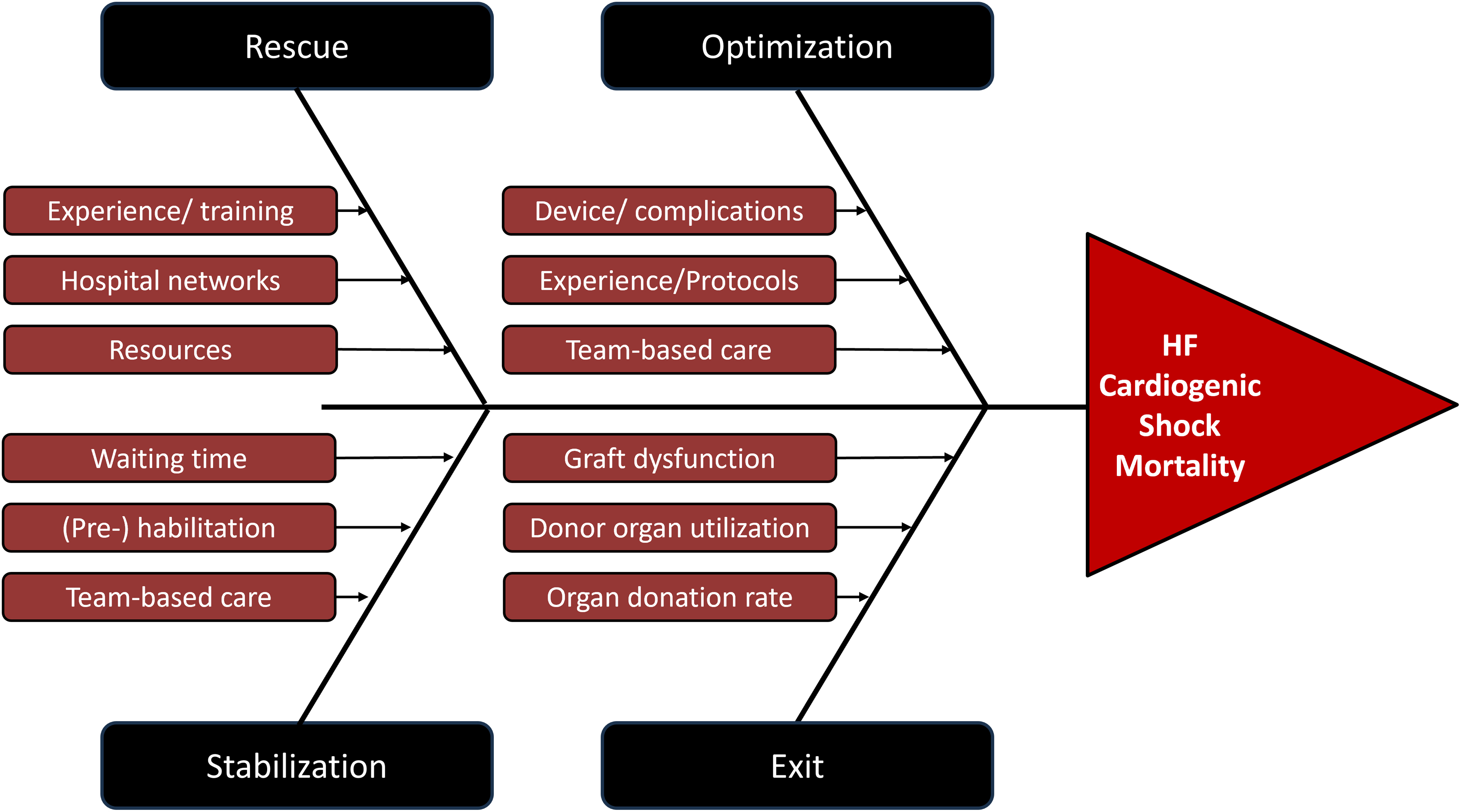

Supplement: Supplementary file 2 — Supplementary material [file mmc2.jpg]
